# Aryl hydrocarbon receptor (AHR)-regulated transcriptomic changes in rats sensitive or resistant to major dioxin toxicities

**DOI:** 10.1186/1471-2164-11-263

**Published:** 2010-04-26

**Authors:** Ivy D Moffat, Paul C Boutros, Hanbo Chen, Allan B Okey, Raimo Pohjanvirta

**Affiliations:** 1 Department of Pharmacology and Toxicology, University of Toronto, Toronto, Canada; 2 Department of Food Hygiene and Environmental Health, Faculty of Veterinary Medicine, University of Helsinki, Helsinki, Finland; 3 National Institute for Health and Welfare, Laboratory of Toxicology, Kuopio, Finland; 4 Ontario Institute for Cancer Research, Toronto, Canada

## Abstract

**Background:**

The major toxic effects of 2,3,7,8-tetrachlorodibenzo-*p*-dioxin (TCDD) appear to result from dysregulation of mRNA levels mediated by the aryl hydrocarbon receptor (AHR). Dioxin-like chemicals alter expression of numerous genes in liver, but it remains unknown which lie in pathways leading to major toxicities such as hepatotoxicity, wasting and lethality. To identify genes involved in these responses we exploited a rat genetic model. Rats expressing an AHR splice-variant lacking a portion of the transactivation domain are highly resistant to dioxin-induced toxicities. We examined changes in hepatic mRNA abundances 19 hours after TCDD treatment in two dioxin-resistant rat strains/lines and two dioxin-sensitive rat strains/lines.

**Results:**

Resistant rat strains/lines exhibited fewer transcriptional changes in response to TCDD than did rats with wildtype AHR. However, well-known AHR-regulated and dioxin-inducible genes such as *CYP1A1*, *CYP1A2*, and *CYP1B1 *remained fully responsive to TCDD in all strains/lines. Pathway analysis indicated that the genes which respond differently to TCDD between sensitive and resistant rats are mainly involved in lipid metabolism, cellular membrane function and energy metabolism. These pathways previously have been shown to respond differently to dioxin treatment in dioxin-sensitive versus dioxin-resistant rats at a biochemical level and in the differential phenotype of toxicologic responses.

**Conclusion:**

The transactivation-domain deletion in dioxin-resistant rats does not abolish global AHR transactivational activity but selectively interferes with expression of subsets of genes that are candidates to mediate or protect from major dioxin toxicities such as hepatotoxicity, wasting and death.

## Background

Dioxin-like chemicals are exceptionally toxic to a wide variety of birds, fish and mammals including, perhaps, humans. However, susceptibility to dioxin toxicity varies widely among different animal species and between genetic types within a species. Extensive evidence demonstrates that virtually all toxic effects of TCDD and related dioxin-like compounds are mediated by a ligand-dependent transcription factor, the aryl hydrocarbon receptor (AHR) (reviewed in: [1]). Exposure to dioxins results in major toxicities, including thymic atrophy, teratogenesis, hepatotoxicity, wasting syndrome and death. These toxicities are dependent on both the AHR and its dimerization partner, the aryl hydrocarbon receptor nuclear translocator (ARNT), and require that the AHR have functional structures for nuclear translocation and DNA binding [2-6].

Dioxin binding converts the AHR into an activated ligand:AHR:ARNT complex that regulates transcription either by binding directly to AHRE-I motifs (also known as DREs or XREs) [7-10] or indirectly to AHRE-II motifs [11,12]. Dioxin toxicities appear to arise from AHR-mediated dysregulation of specific genes [4,13]. Microarray technologies have accelerated identification of genes that depend on the AHR for constitutive expression or for response to TCDD *in vivo *[14-23], but the key genes whose dysregulation by dioxin leads to most toxicities remain unknown.

The Han/Wistar(*Kuopio*) (H/W) rat is an excellent model organism to identify specific AHR-regulated genes whose dysregulation by dioxin may lead to major toxicities. H/W rats are extraordinarily resistant to acute lethality from TCDD, with an LD_50 _three orders of magnitude higher than for sensitive Long-Evans(*Turku/AB*) rats (L-E) [24]. Resistance in H/W rats is associated with a point mutation that leads to expression of an aberrant AHR protein missing 38 or 43 amino acids from its transactivation domain (TAD) [25-27]. We recently demonstrated, using transgenic mouse models, that it is the AHR variant with 38 amino acids deleted that is responsible for dioxin resistance [28]. Importantly, this AHR variant is the predominantly-expressed form in the dioxin-resistant H/W rat [27]. Further, dioxin resistance in rats segregates genetically with the *AHR *locus and is a dominant trait [24,26]. Multi-generational crosses of L-E and H/W rats and selection for susceptibility or resistance to dioxin lethality [26] produced two rat lines: Line-A (LnA) and Line-C (LnC). LnA rats harbour the variant AHR and are dioxin-resistant; LnC rats have the wildtype AHR and are dioxin-sensitive. We postulate that the partial deletion of AHR transactivation domain alters toxic responses either by preventing changes in mRNA levels of genes in pro-death pathways or by enhancing responses of genes in pro-survival pathways.

Our strategy for identifying pro-survival or pro-death genes is to contrast changes in mRNA expression profiles following TCDD exposure of dioxin-sensitive and dioxin-resistant rats [13]. To reduce the influence of strain-specific changes not associated with dioxin toxicities, we profiled 4 strains/lines of rats: H/W and LnA which constitute the "resistant collective" and L-E and LnC which constitute the "sensitive collective".

We focused on hepatic mRNA levels because liver displays a broad spectrum of mRNAs that are responsive to dioxins and/or to *AHR *genotype [20] and because liver is a prime site of dioxin toxicity, displaying many phenotypic differences between sensitive and resistant rats [29]. We chose a dose of 100 μg/kg TCDD, which produces hepatotoxicity, wasting and death in sensitive rats but no deaths in resistant rats. We previously conducted a smaller-scale transcriptomic study in sensitive versus resistant rats on membrane arrays and using cross-species hybridization to cDNA arrays [30]. Here, we greatly extend those prior studies by assessing transcriptome-wide responses to TCDD which were further validated via real-time RT-PCR. We identify specific biological processes perturbed by TCDD exposure.

Our analysis paints a new picture of dioxin-induced expression changes. Hundreds of genes exhibit responses to TCDD that are specific to individual strains or lines. Our genetic model diminishes this background noise and identifies a small number of genes associated with hepatotoxicity, wasting and death. Genes differentially-expressed between sensitive and resistant strains show functional homogeneity: dioxin-lethality may be associated with broad dysregulation of entire pathways, not just single genes.

## Results

**Figure 1:**
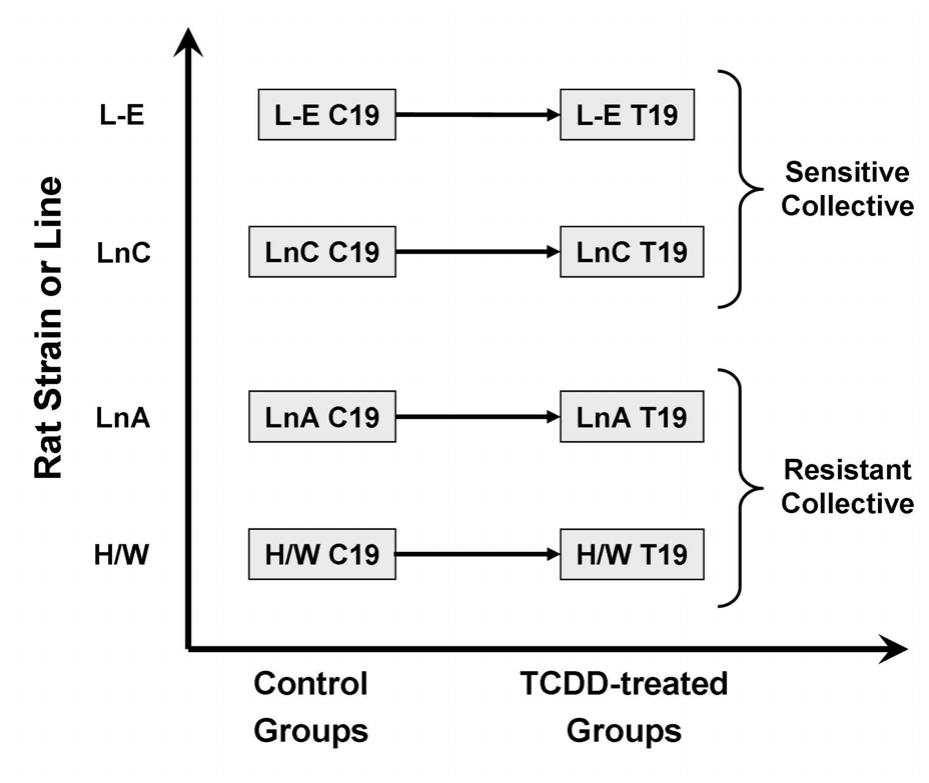
**Experimental Design**. A two-factor design was used to assess the effects of strain/line (L-E, LnC, LnA and H/W) and TCDD-exposure (control or 19-hour exposure to 100 μg/kg TCDD). In total 32 mRNA profiles were assessed by individual RAE230A microarrays; four separate animals were profiled for each of the eight separate experimental conditions.

To determine the effect of TCDD on mRNA abundances in dioxin-sensitive rats versus dioxin-resistant rats we studied four rat strains/lines at 19 hours after oral administration of TCDD (Figure 1) using Affymetrix RAE230A microarrays. A list of all genes and their responses to TCDD in the four rat strains/lines is given in Additional File 1.

### Global differences in mRNA abundance

**Figure 2:**
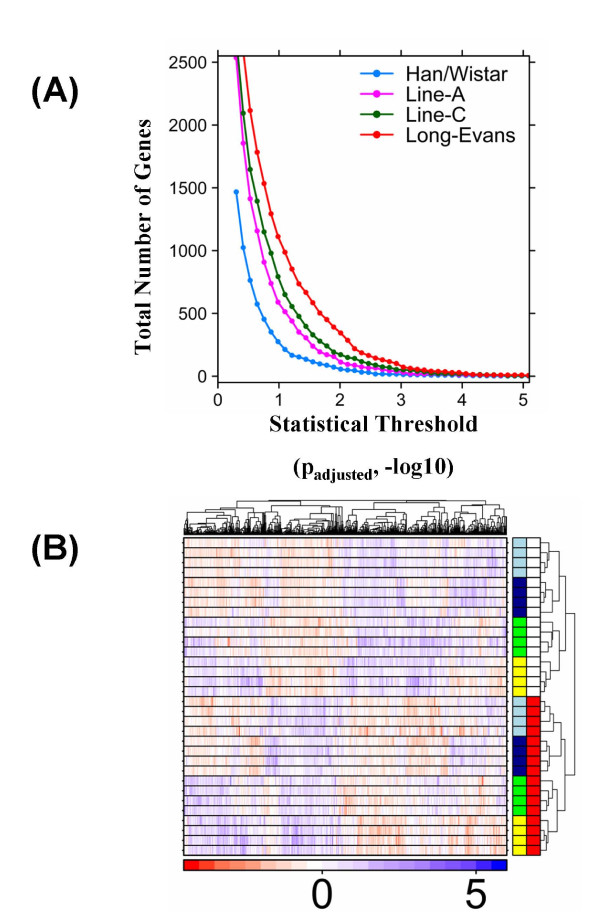
**Global comparison of expression profiles between rat strains/lines**. **(A) **The total number of genes affected by TCDD treatment in each rat line or strain is compared at levels of statistical significance (adjusted p-values; -log10) ranging from 0 to 5. **(B) **Hierarchical clustering with within-row scaling of all non-constant genes (variance > 0.1). Within the heatmap, blue indicates genes induced (up-regulated) by TCDD; red indicates genes repressed (down-regulated) by TCDD. Within the annotation bars (right side of heatmap), red indicates which rats were exposed to TCDD and white indicates those exposed to corn oil vehicle. The first column of annotation bars indicates the strains/lines of animals profiled: yellow, L-E; light blue, LnC; green, H/W, dark blue, LnA. The colour-scale gives within-row-scaled expression values, with red hues indicating low-expression and blue hues indicating high-expression.

The total number of genes affected by TCDD varied across rat strains/lines in a manner independent of the statistical threshold (Figure 2A). More genes were affected by TCDD in dioxin-sensitive rats than in dioxin-resistant rats: across all four strains/lines, the order of the number of transcriptional alterations was: L-E > LnC > LnA > H/W. Overall, it appears that the deletion in the AHR transactivation domain reduces the number of genes altered but does not ablate the transcriptional response to TCDD.

Unsupervised clustering analysis (Figure 2B) using all non-constant genes (variance > 0.01) revealed the strongest trend in the dataset was the distinction between TCDD-treated (red annotation bars) and vehicle-treated (white annotation bars) animals. The co-clustering of the two F_2 _crosses (LnA, dark blue; LnC, light blue) may indicate that their common parentage is a stronger determinant of their transcriptional profiles than is their sensitivity or resistance to TCDD toxicity. Further, this suggests that number of genes causally related to the hepatotoxicity (and possibly acute lethality) of TCDD is small compared with all other changes caused by TCDD in hepatic gene expression.

**Figure 3:**
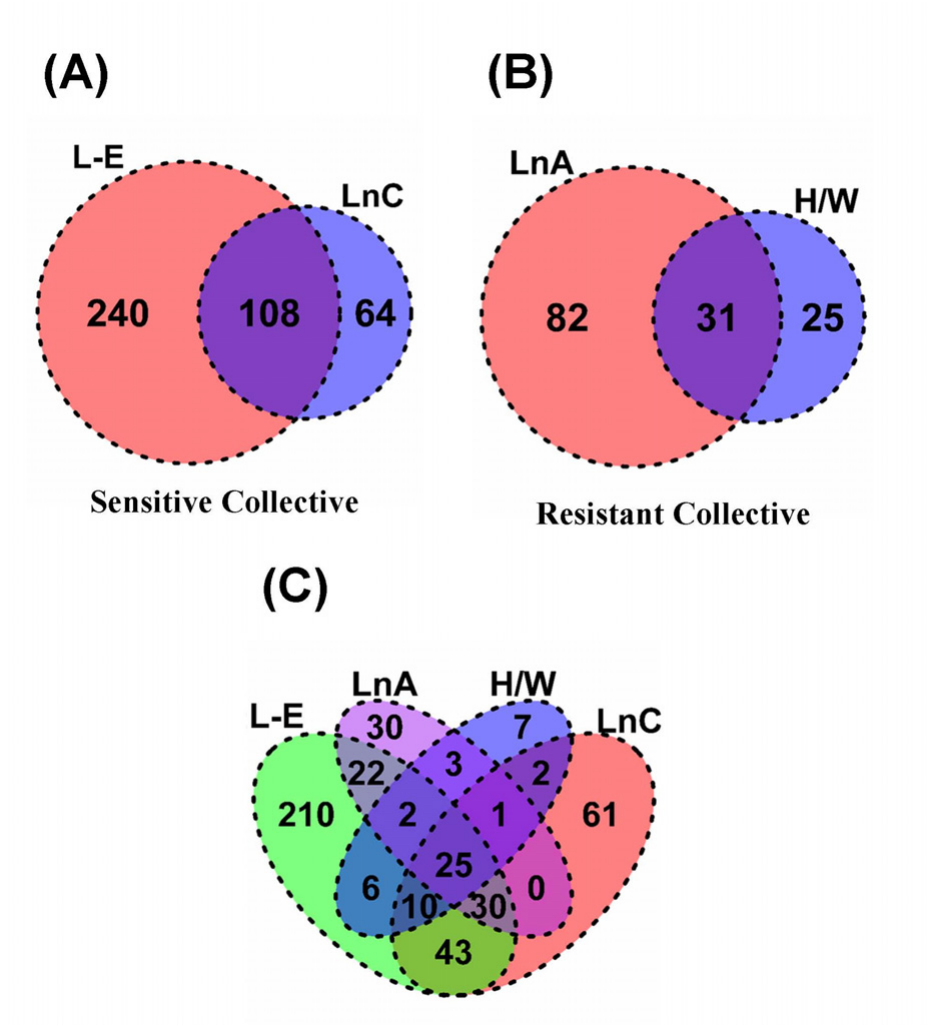
**Overlap of altered transcripts between rat strains/lines**. We generated Venn diagrams to visualize the overlap of significantly (p_adjusted _< 0.01) altered transcript responses to 100 μg/kg TCDD for 19 hours between the sensitive and resistant collectives: **(A) **across the sensitive collective, **(B) **across the resistant collective and **(C) **across all four strains/lines.

The extent of the overlap in transcriptional responses to TCDD among sensitive and resistant rat strains/lines was visualized using a two-way table (Table 1) and Venn diagrams (Figure 3). Of the 8605 genes interrogated, 452 (5.3%) responded in at least one strain or line at a 1% false-discovery rate; 8153 genes did not respond to TCDD in any strain/line (Table 1). Of the 452 responsive genes only 144 (31.9%) were altered in more than one strain/line. Only 25 genes responded in all strains/lines, of which 20 were induced while only 5 were genes repressed. This direction of response is concordant with our previous finding that 70% of genes altered in common by TCDD in both mouse and rat are up-regulated [23].

**Table 1:** Extent of overlap in transcript responses to TCDD among dioxin-sensitive and dioxin-resistant rat strains/lines

		Sensitive Collective Score				
		
		-2	-1	0	1	2
**Resistant Collective Score**	**-2**	5	1	1	0	0
	
	**-1**	14	18	7	0	0
	
	**0**	26	177	8153	94	17
	
	**1**	0	0	30	12	26
	
	**2**	0	0	2	2	20

To evaluate the overlap of significantly (p_adjusted _< 0.01) altered transcripts between the sensitive and resistant collectives we formed a two-way table. A TCDD-responsive score of -1 (repressed), 0 (unchanged), or +1 (induced) was assigned to each gene in each strain. The sum of the scores for the sensitive strains/lines form the columns and the sum of the scores for the resistant strains/lines are the rows. Each value corresponds to the number of genes that exhibited a significant response to TCDD treatment. For example, 20 genes are induced in both sensitive and both resistant strains/lines, while 5 genes are repressed in both of the sensitive strains/lines and both of the resistant strains/lines. Of the 8605 genes examined, 452 genes responded in at least 1 strain/line, while 8153 genes did not respond to TCDD in any rat strain/line.

The sensitive collective exhibited a greater number of responsive genes (412) and a greater overlap of these genes among strains/lines within the collective (Figure 3A) than did the resistant collective (138 responsive genes; Figure 3B). However, the proportion of TCDD-responsive genes that overlapped within each collective to the total genes on the array that responded to TCDD in that collective did not differ between collectives (22.5% in the resistant vs. 26.2% in the sensitive collective, p = 0.44; proportion test).

### Classification of Type-I vs. Type-II responses to TCDD

Identifying which of the 452 TCDD-responsive genes are most likely to be involved in major dioxin toxicities is challenging; we therefore exploited our genetic model. Each TCDD-responsive gene was classified according to the Type-I/Type-II TCDD response scheme previously developed for toxic endpoints [31].

Type-I responses to TCDD are those that are similar between dioxin-sensitive rat strains/lines and dioxin-resistant rat strains/lines. We considered genes that exhibited a statistically significant response to TCDD in all four strains/lines to be Type-I genes. By this definition 25 genes were classified as Type-I (Figure 3C; Score ± 4 in Table 1). These Type-I genes include well-known (Table 2) as well as novel TCDD-responsive genes (Additional File 1). The vast majority of Type-I genes (20/25) were up-regulated by TCDD (score +4 in Table 1). For some Type-I genes the magnitude of induction was very large, including ~90-fold induction of *CYP1A1 *mRNA and ~75-fold induction of *ALDH3A1 *mRNA.

**Table 2:** Known dioxin-inducible genes

Resistant Collective				Sensitive Collective						
			
H/W		LnA		L-E		LnC				
**FC log_2_**	**p_adj_**	**FC log_2_**	**p_adj_**	**FC log_2_**	**p_adj_**	**FC log_2_**	**p_adj_**	**Gene ID**	**Symbol**	**Full Name**

6.3	3.2 × 10^-9^	5.4	8.2 × 10^-8^	6.8	2.2 × 10^-12^	6.2	8.9 × 10^-10^	25375	*Aldh3a1*	aldehyde dehydrogenase 3 family, member A1
6.4	1.3 × 10^-4^	6.1	1.0 × 10^-6^	6.6	3.3 × 10^-8^	6.4	1.8 × 10^-9^	24296	*Cyp1a1*	cytochrome P450, family 1, subfamily a, polypeptide 1
1.2	2.0 × 10^-5^	1.2	1.6 × 10^-5^	1.1	1.5 × 10^-6^	1.1	1.9 × 10^-5^	24297	*Cyp1a2*	cytochrome P450, family 1, subfamily a, polypeptide 2
3.9	6.8 × 10^-5^	3.5	4.9 × 10^-7^	5.3	4.4 × 10^-6^	4.9	7.8 × 10^-6^	25426	*Cyp1b1*	cytochrome P450, family 1, subfamily b, polypeptide 1
1.2	4.4 × 10^-3^	1.1	1.9 × 10^-3^	1.9	3.8 × 10^-4^	1.4	1.9 × 10^-3^	83619	*Nfe2l2*	nuclear factor, erythroid derived 2, like 2
3.7	3.2 × 10^-5^	2.5	1.4 × 10^-4^	2.4	4.9 × 10^-5^	2.6	4.5 × 10^-5^	24314	*Nqo1*	NAD(P)H dehydrogenase, quinone 1
2.3	1.2 × 10^-3^	1.8	1.3 × 10^-3^	2.5	1.6 × 10^-4^	2.2	1.7 × 10^-4^	310467	*Tiparp*	TCDD-inducible poly(ADP-ribose) polymerase

Transcriptional responses after 19-hour TCDD exposure in the four rat strains/lines were identified using Affymetrix RAE230A arrays followed by data pre-processing and statistical testing using linear models. Twenty-five Type-I genes (similar response to TCDD in all four strains/lines) were identified. These included several well-established AHR-regulated and dioxin-inducible genes, supporting the validity of the array experiments. For each strain/line, the fold-change (FC in log_2 _space) in mRNA levels between treated and control rats as well as the significance levels are presented.

Type-II responses to TCDD are those that differ between dioxin-sensitive rats and dioxin-resistant rats. Genes that responded to TCDD exposure in a statistically significant manner in both members of one collective but neither of the strains/lines in the other collective are classified as Type-II genes. By this classification, 46 genes exhibited Type-II responses (Figure 3C; Score ± 2 & 0 in both ways in Table 1). Of these, only three were altered in resistant rats but not in sensitive rats (*Il1r1*, *Phyh*, *Hacl1*). The remaining 43 genes were specifically altered only in dioxin-sensitive rats. In contrast to the 80% upregulation of Type-I genes, 61% (28/46) of Type-II genes were downregulated by TCDD.

### Validation by real-time RT-PCR

The validity of our array experiments to identify effects of TCDD on mRNA levels is supported by the facts that well-established dioxin-inducible genes were identified (e.g. *CYP1A1*, *CYP1A2, CYP1B1 *and *Tiparp*; Table 2) and there is overlap between our current list of responsive genes and genes previously reported to be affected by AHR-ligands in other array experiments [15,17].

**Figure 4:**
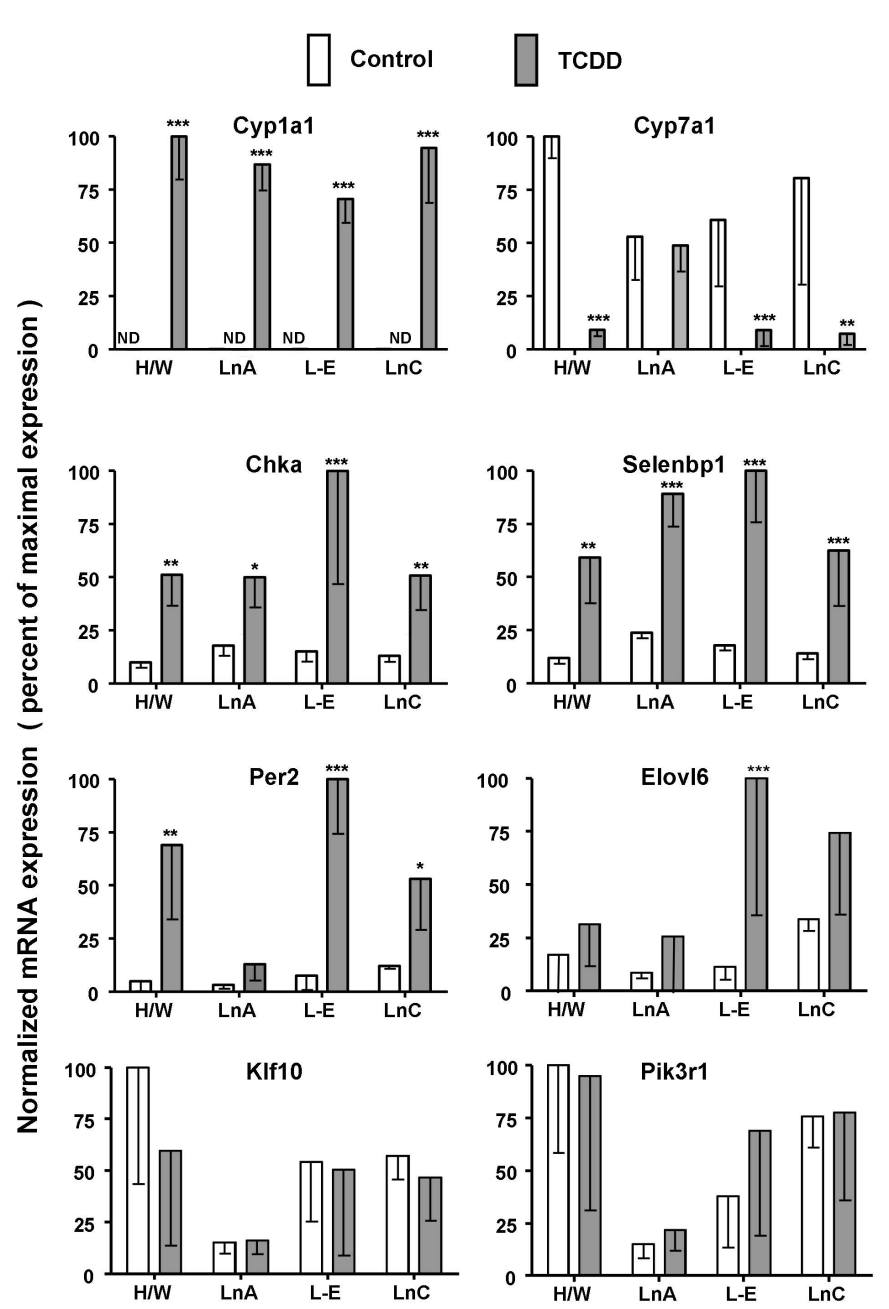
**Gene responses to TCDD exposure in livers of dioxin-resistant and dioxin-sensitive rats: measurement of selected mRNA levels by real-time RT-PCR**. Hepatic RNA was prepared from male adult TCDD-sensitive rats (L-E and LnC) and TCDD-resistant rats (H/W and LnA) after 19-hour treatment with a single dose of 100 μg/kg TCDD or corn-oil vehicle control by gavage. mRNA levels were measured by real-time RT-PCR and normalized as described in Materials & Methods. For each gene, the mRNA level that was highest for any strain/line or treatment was set at 100% and all other mRNA levels for that gene are shown as a percentage of that maximal level. All results plotted represent the mean ± standard deviation of four rats. Asterisks indicate significant differences in mRNA levels between control and TCDD-treated rats (t-test; two-tailed, unequal variance, * p < 0.05, ** p < 0.01, *** p < 0.001). Note: levels of CYP1A1 mRNA in control animals were below detection limits (ND).

To further assess the validity of our array results we employed real-time RT-PCR to evaluate effects of TCDD on mRNA levels. Genes were selected for RT-PCR analysis to span a wide range of magnitudes of response (fold-change in log_2_: high > 4; medium 2-4; low < 2; or no response). To this end, both array (Table 2) and RT-PCR analyses (Figure 4) showed a high magnitude of TCDD-mediated induction of the prototypical responsive/AHR-activated gene *CYP1A1 *[13,32,33]. Further, *CYP7A1* and *Chka* exhibited medium magnitudes of response to TCDD in both collectives as evidenced by RT-PCR assays (Figure 4) as well as, array experiments (Additional File 1). *Selenbp1* and *Per2* showed low magnitudes of TCDD-mediated induction in both collectives as evidenced by array experiments (Additional File 1) and slightly higher magnitudes of induction by RT-PCR assays (Figure 4). *Elov6* exhibited significant induction in the dioxin-sensitive collective but not in the resistant collective as assayed both by array (Table 3) and RT-PCR (Figure 4). TCDD had no significant effect on mRNA levels for *Klf10* or *Pik3r1* as assayed either by RT-PCR (Figure 4) or by gene array (Additional File 1).

**Table 3:** Type-II gene responses 19 hours after TCDD exposure

Resistant Collective				Sensitive Collective						
			
H/W		LnA		L-E		LnC				
**FC log_2_**	**p_adj_**	**FC log_2_**	**p_adj_**	**FC log_2_**	**p_adj_**	**FC log_2_**	**p_adj_**	**Gene ID**	**Symbol**	**Full Name**

-0.3	7.3 × 10^-2^	-0.4	1.1 × 10^-2^	-0.7	5.7 × 10^-4^	-0.3	3.0 × 10^-3^	25368	*Adk*	adenosine kinase
0.4	2.2 × 10^-2^	0.2	5.6 × 10^-2^	0.6	6.2 × 10^-3^	0.4	4.8 × 10^-3^	305338	*Apbb2*	amyloid beta (A4) precursor protein-binding, family B, member 2
-0.4	1.4 × 10^-1^	-0.3	6.8 × 10^-2^	-0.8	1.5 × 10^-3^	-0.5	2.9 × 10^-3^	25698	*Ass1*	argininosuccinate synthetase 1
0.3	7.3 × 10^-2^	0.4	1.1 × 10^-2^	0.4	7.3 × 10^-3^	0.4	1.4 × 10^-3^	116550	*Atp5c1*	ATP synthase, H+ transporting, mitochondrial F1 complex, gamma polypeptide 1
-0.9	6.5 × 10^-2^	-0.6	4.8 × 10^-2^	-0.9	4.3 × 10^-3^	-0.8	4.5 × 10^-3^	64828	*B4galnt1*	beta-1,4-N-acetyl-galactosaminyl transferase 1
0.3	1.6 × 10^-1^	0.2	6.0 × 10^-2^	0.6	4.3 × 10^-3^	0.7	1.5 × 10^-3^	113948	*Bbs2*	Bardet-Biedl syndrome 2 homolog (human)
0.3	3.0 × 10^-1^	0.5	5.7 × 10^-2^	0.5	1.0 × 10^-3^	0.6	2.2 × 10^-3^	292027	*Cfdp1*	craniofacial development protein 1
-0.2	2.3 × 10^-1^	-0.3	7.1 × 10^-2^	-0.6	1.0 × 10^-3^	-0.6	1.5 × 10^-3^	85251	*Col18a1*	collagen, type XVIII, alpha 1
-0.3	3.2 × 10^-1^	-0.5	6.6 × 10^-2^	-0.9	1.7 × 10^-4^	-0.4	9.4 × 10^-3^	266682	*Cyp3a2*	cytochrome P450, family 3, subfamily a, polypeptide 2
-0.4	2.8 × 10^-1^	-0.5	3.3 × 10^-2^	-0.9	7.8 × 10^-5^	-0.5	2.2 × 10^-3^	286904	*Cyp4f4*	cytochrome P450, family 4, subfamily f, polypeptide 4
0.4	1.2 × 10^-2^	0.2	5.0 × 10^-2^	0.4	1.2 × 10^-3^	0.4	3.5 × 10^-3^	362912	*Derl1*	Der1-like domain family, member 1
0.6	6.6 × 10^-2^	0.3	2.3 × 10^-2^	0.6	1.7 × 10^-3^	0.6	6.5 × 10^-4^	691956	*Derl2*	Der1-like domain family, member 2
-0.4	1.5 × 10^-1^	-0.3	1.0 × 10^-1^	-0.4	5.9 × 10^-3^	-0.5	9.7 × 10^-3^	25313	*Egf*	epidermal growth factor
0.3	6.3 × 10^-1^	1.1	2.0 × 10^-1^	1.2	3.3 × 10^-4^	0.6	9.8 × 10^-3^	171402	*Elovl6*	ELOVL family member 6, elongation of long chain fatty acids (yeast)
-0.3	2.9 × 10^-1^	-0.3	7.2 × 10^-2^	-0.7	1.0 × 10^-3^	-0.5	9.4 × 10^-3^	29580	*Fdft1*	farnesyl diphosphate farnesyl transferase 1
-0.3	3.7 × 10^-1^	-0.3	4.3 × 10^-2^	-0.7	4.2 × 10^-4^	-0.4	4.1 × 10^-3^	297029	*Gstk1*	glutathione S-transferase kappa 1
-0.5	9.3 × 10^-2^	-0.2	1.8 × 10^-1^	-0.7	6.9 × 10^-3^	-0.4	3.0 × 10^-3^	81869	*Gstm7*	glutathione S-transferase, mu 7
-1.3	7.2 × 10^-3^	-1.0	1.1 × 10^-3^	-1.1	3.4 × 10^-2^	-1.2	1.2 × 10^-2^	85255	*Hacl1*	2-hydroxyacyl-CoA lyase 1
-0.2	6.3 × 10^-1^	-0.3	1.8 × 10^-1^	-0.7	6.5 × 10^-4^	-0.7	2.2 × 10^-3^	24439	*Hagh*	hydroxyacyl glutathione hydrolase
-0.6	2.5 × 10^-2^	-0.2	2.1 × 10^-1^	-0.6	2.7 × 10^-3^	-0.7	4.7 × 10^-3^	25116	*Hsd11b1*	hydroxysteroid 11-beta dehydrogenase 1
0.5	1.5 × 10^-1^	0.3	1.1 × 10^-1^	0.9	1.0 × 10^-3^	0.6	4.8 × 10^-3^	84013	*Hsd17b12*	hydroxysteroid (17-beta) dehydrogenase 12
1.4	5.1 × 10^-3^	0.5	3.9 × 10^-3^	0.7	4.3 × 10^-2^	0.9	1.5 × 10^-2^	25663	*Il1r1*	interleukin 1 receptor, type I
-0.6	3.7 × 10^-2^	-0.3	1.8 × 10^-1^	-0.7	3.8 × 10^-3^	-0.8	6.1 × 10^-4^	690745	*LOC690745*	MOCO sulphurase C-terminal domain containing-like
0.2	8.5 × 10^-1^	-0.5	4.2 × 10^-2^	-0.8	8.5 × 10^-4^	-0.5	4.8 × 10^-3^	497794	*Mug1*	murinoglobulin 1
-0.4	8.6 × 10^-2^	-0.4	4.1 × 10^-2^	-0.6	3.6 × 10^-3^	-0.6	8.6 × 10^-3^	29227	*Nfib*	nuclear factor I/B
-0.5	9.3 × 10^-2^	-0.6	3.2 × 10^-2^	-1.2	1.0 × 10^-3^	-0.5	1.4 × 10^-3^	680451	*Nrbp2*	nuclear receptor binding protein 2
-0.2	6.1 × 10^-1^	-0.4	1.0 × 10^-2^	-0.6	2.5 × 10^-4^	-0.5	3.4 × 10^-3^	94267	*Nudt4*	nudix (nucleoside diphosphate linked moiety X)-type motif 4
1.0	1.6 × 10^-2^	0.7	4.2 × 10^-2^	0.7	4.6 × 10^-3^	1.2	1.9 × 10^-5^	171564	*Pbld*	phenazine biosynthesis-like protein domain containing
0.4	4.5 × 10^-2^	0.3	4.2 × 10^-2^	0.4	7.6 × 10^-3^	0.4	1.5 × 10^-3^	308061	*Pdcd6*	programmed cell death 6
0.7	5.1 × 10^-3^	0.7	2.0 × 10^-4^	0.4	1.1 × 10^-1^	0.7	1.3 × 10^-2^	114209	*Phyh*	phytanoyl-CoA 2-hydroxylase
0.6	1.6 × 10^-2^	0.4	5.2 × 10^-2^	0.5	2.7 × 10^-3^	0.8	7.7 × 10^-5^	64390	*Prpsap1*	phosphoribosyl pyrophosphate synthetase-associated protein 1
-0.3	3.3 × 10^-1^	-0.4	7.0 × 10^-2^	-0.6	4.3 × 10^-3^	-0.5	2.9 × 10^-3^	315655	*Rdx*	radixin
-0.2	1.2 × 10^-1^	-0.4	1.5 × 10^-2^	-0.6	5.0 × 10^-3^	-0.5	1.3 × 10^-3^	363160	*RGD1311563*	similar to Oligosaccharyl transferase 3 CG7748-PA
0.1	3.1 × 10^-1^	0.2	1.1 × 10^-1^	1.4	7.3 × 10^-3^	0.7	4.1 × 10^-4^	315611	*Scn4b*	sodium channel, type IV, beta
-0.3	3.7 × 10^-1^	-0.6	2.9 × 10^-2^	-1.2	6.8 × 10^-4^	-0.9	5.1 × 10^-3^	25216	*Sdc1*	syndecan 1
0.4	8.4 × 10^-2^	0.4	2.3 × 10^-2^	0.5	4.3 × 10^-3^	0.6	7.9 × 10^-3^	680891	*Sf3b5*	splicing factor 3b, subunit 5
-0.2	5.9 × 10^-1^	-0.3	3.5 × 10^-2^	-0.5	2.5 × 10^-4^	-0.4	2.6 × 10^-3^	81536	*Sgpp1*	sphingosine-1-phosphate phosphatase 1
-0.8	2.1 × 10^-2^	-0.6	5.0 × 10^-2^	-0.7	4.2 × 10^-4^	-0.7	2.0 × 10^-3^	79111	*Slc27a5*	solute carrier family 27 (fatty acid transporter), member 5
0.6	2.1 × 10^-2^	0.4	1.7 × 10^-2^	0.5	4.4 × 10^-3^	0.7	3.7 × 10^-4^	500707	*Tc2n*	tandem C2 domains, nuclear
0.2	4.4 × 10^-1^	0.3	5.0 × 10^-2^	0.3	7.0 × 10^-3^	0.5	3.0 × 10^-3^	367909	*Tceal8*	transcription elongation factor A (SII)-like 8
-0.2	3.7 × 10^-1^	-0.3	8.3 × 10^-2^	-0.5	3.3 × 10^-3^	-0.4	3.4 × 10^-3^	170907	*Tpo1*	developmentally regulated protein TPO1
0.7	2.2 × 10^-2^	0.3	7.4 × 10^-2^	0.6	7.5 × 10^-3^	0.6	4.1 × 10^-3^	362890	*Tspan31*	tetraspanin 31
-0.2	3.8 × 10^-1^	-0.1	7.8 × 10^-1^	-0.4	6.3 × 10^-3^	-0.4	3.4 × 10^-3^	362696	*Ttc7*	tetratricopeptide repeat domain 7
-0.1	7.8 × 10^-1^	-0.3	6.2 × 10^-2^	-0.5	5.4 × 10^-3^	-0.5	4.5 × 10^-3^	363869	*Ubl3*	ubiquitin-like 3
-0.9	1.5 × 10^-1^	-0.5	2.9 × 10^-1^	-0.9	1.5 × 10^-4^	-1.1	1.3 × 10^-3^	117522	*Xpnpep2*	X-prolyl aminopeptidase (aminopeptidase P) 2, membrane-bound
0.3	4.6 × 10^-2^	0.4	1.2 × 10^-2^	0.6	1.3 × 10^-3^	0.7	4.3 × 10^-3^	360389	*Zfp422*	zinc finger protein 422

Transcriptional responses after 19 hours TCDD exposure in the four rat strains/lines were identified using Affymetrix RAE230A arrays followed by data pre-processing and statistical testing using linear models. Type-II genes were identified as those where both strains/lines in one collective showed statistically significant responses to TCDD (p_adjusted _< 0.01) in the same direction, while both strains/lines in the other collective showed no response (p_adjusted _> 0.01). For each strain/line, the fold-change (FC in log_2 _space) in mRNA levels between treated and control rats as well as the significance levels are presented. Genes are sorted alphabetically by gene symbol.

### Analysis of the AHR role in regulation

**Figure 5:**
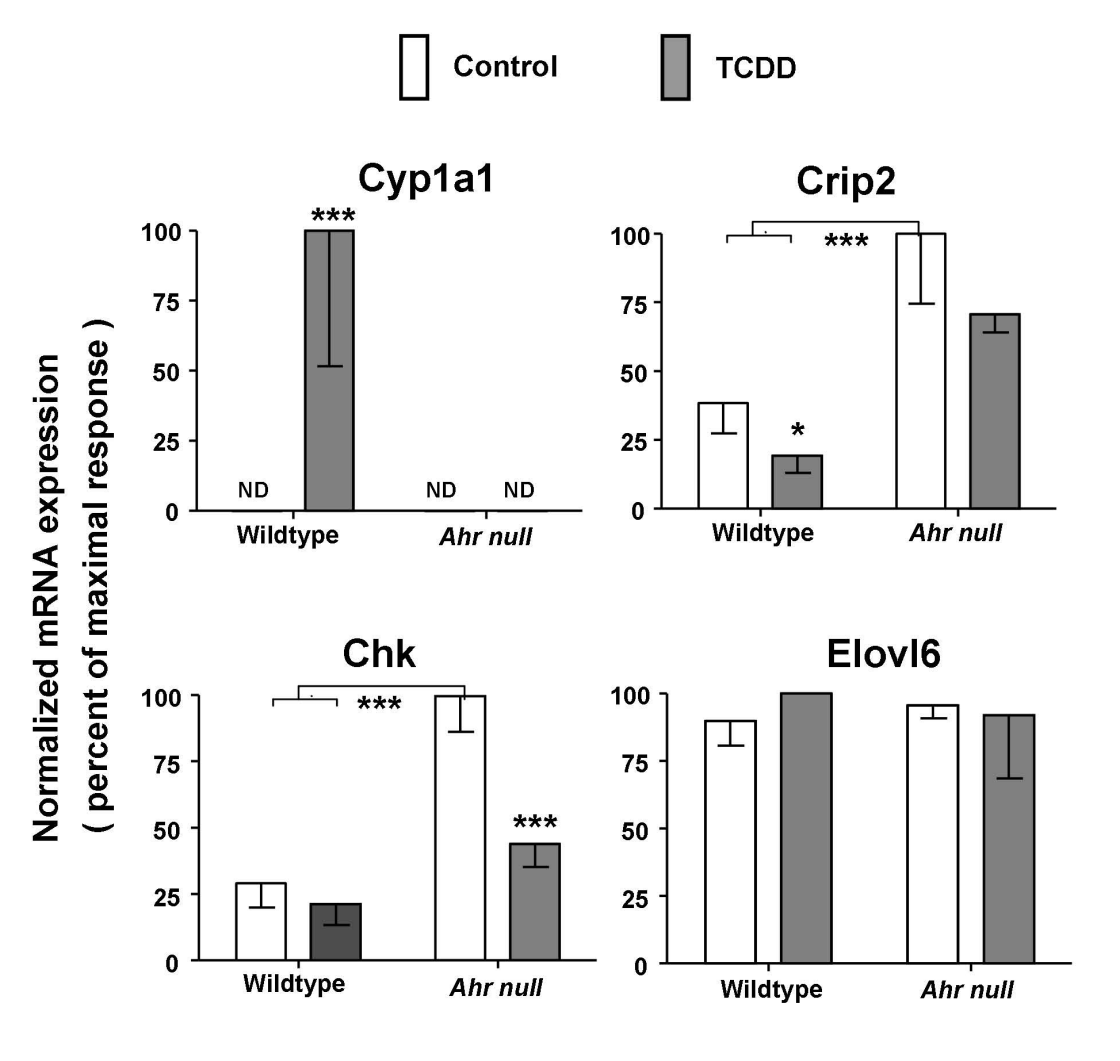
**Gene responses to TCDD exposure in livers of Ahr-null versus wildtype mice: measurement of selected mRNA levels by real-time RT-PCR**. Hepatic RNA was prepared, as described in Materials and Methods, from male adult *Ahr*-null mice (*Ahr*^-/-^) and wildtype C57BL/6J mice (*Ahr*^+/+^) after treatment with a single dose of 1000 μg/kg TCDD or corn oil vehicle for 19 hours. There were 3 TCDD-treated and 3 control mice in the *Ahr*-null groups and 4 TCDD-treated and 4 control mice in the wildtype groups. Levels for selected mRNAs used in the RT-PCR array validity experiments in rats were measured by real-time RT-PCR and normalized to Actb in this mouse model [60]. For each gene, the highest mRNA level across all experiments was set at 100% and all other mRNA levels for that gene are shown as a percentage of that maximal level. Error bars represent standard deviation of the mean. Asterisks indicate differences in mRNA levels (ANOVA followed by Bonferroni *post hoc *tests, * p < 0.05, *** p < 0.001). Note that for CYP1A1 the mRNA level in control animals or in TCDD-treated *Ahr*^-/- ^mice or control *Ahr*^-/- ^mice is below the detection limit of the assay; thus there are no bars visible for these groups in this plot (ND).

It is well-established that major toxicities of TCDD require the AHR [2,4,5,34,35]. To determine if the AHR was required for the gene to respond to TCDD we compared hepatic mRNA levels for a few genes in *Ahr*-null mice (*Ahr^-/-^*) versus mice with wildtype AH receptor (*Ahr^+/+^*). Mice were treated with a dose of TCDD equitoxic to that in sensitive rats [23] for a comparable time. As expected, induction of CYP1A1 mRNA was strictly dependent on the AHR (Figure 5). Suppression of *Crip2* mRNA levels also was AHR-dependent (Figure 5). Regulation of *Chka* and *Elovl6* by TCDD appears to be species-specific: *Chka* mRNA was upregulated in rats from both collectives (Additional File 1 and Figure 4) but was significantly downregulated in *Ahr*-null mice and unaffected in wildtype mice (Figure 5); *Elovl6* was upregulated in dioxin-sensitive rats (Table 3 and Figure 4) but *Elovl6* was unresponsive to TCDD in both wildtype and *Ahr*-null mice (Figure 5). The findings with *Chka* and *Elovl6* reinforce recent reports of substantial differences between rat and mouse in transcriptional responses to TCDD [16,23].

### Pathway Analysis

**Figure 6:**
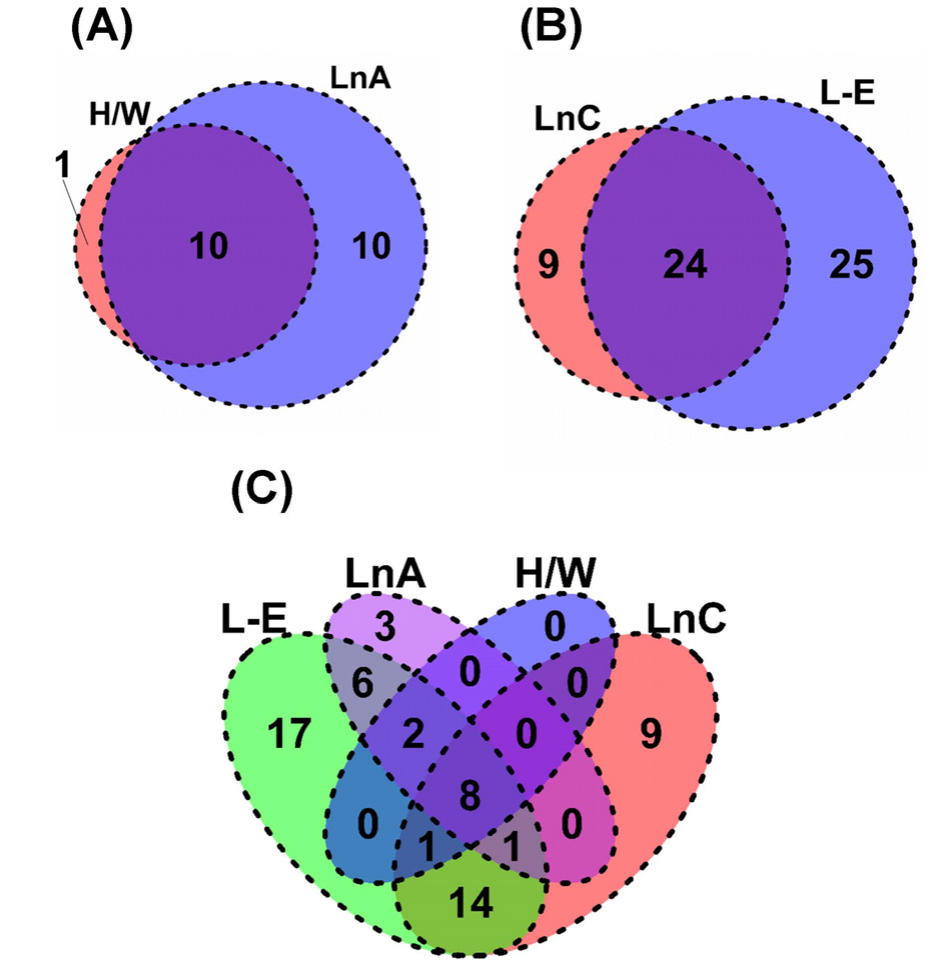
**Overlap of functional terms between rat strains/lines**. Gene Ontology (GO) analysis was used to determine if different combinations of strains/lines led to alterations in mRNA levels for functionally coherent groups of genes. The gene-lists for each of the four strains/lines were tested for enrichment of each GO category represented on the RAE230A array. False-discovery rates were calculated with 1000 permutations of the dataset using the High-Throughput GoMiner software and a threshold of 5% FDR was applied. The vast majority of GO terms enriched in both the resistant (A) and the sensitive (B) strains/lines overlap. A four-way overlap of all strains/lines shows significant overlap, but also some strain-specific responses (C).

To determine if alterations in mRNA abundances caused by TCDD are functionally coherent, we performed two Gene Ontology (GO) analyses. The first used candidate genes from each rat strain/line to determine the extent of overlap of pathways between rat strains/lines. We found that the pathways dysregulated by TCDD were very similar in the two dioxin-resistant rats, LnA and H/W (Figure 6A) and in the two dioxin-sensitive rats, LnC and L-E (Figure 6B). Further, there was overlap in pathways among all strains/lines: 8 GO terms were enriched in all four strains/lines, while 14 GO terms were specifically enriched in only the sensitive strains (Figure 6C).

The second analysis used both Type-I and Type-II gene lists to determine the specific GO terms enriched in each of the Type-I and Type-II gene lists. This analysis identified 5 GO terms - mostly relating to cytochrome P450 genes - enriched in the Type-I genes (Table 4). Amongst the Type-II genes, the analysis identified 14 GO terms specifically enriched (Table 4). In particular, genes related to the endoplasmic reticulum were present about 4 times as often as expected by chance alone, as were genes related to lipid metabolism.

**Table 4:** Enrichment of functional terms within Type-I response and Type-II response gene lists from rat strains/lines

	Type-I		Type-II		
			
GO ID	Enrichment	FDR	Enrichment	FDR	Functional term
GO:0042175	2.20	1.00	**2.68**	**0.00**	nuclear envelope-endoplasmic reticulum network
GO:0005789	2.23	1.00	**2.71**	**0.00**	endoplasmic reticulum membrane
GO:0044432	2.06	1.00	**2.54**	**0.00**	endoplasmic reticulum part
GO:0044255	1.44	1.00	**2.21**	**0.01**	cellular lipid metabolic process
GO:0006629	1.28	1.00	**2.05**	**0.01**	lipid metabolic process
GO:0005783	1.63	1.00	**1.99**	**0.01**	endoplasmic reticulum
GO:0012505	1.07	1.00	**1.84**	**0.02**	endomembrane system
GO:0031090	0.55	1.00	**1.57**	**0.02**	organelle membrane
GO:0031301	NA	1.00	**3.70**	**0.02**	integral to organelle membrane
GO:0031300	NA	1.00	**3.57**	**0.02**	intrinsic to organelle membrane
GO:0030176	NA	1.00	**4.46**	**0.02**	integral to endoplasmic reticulum membrane
GO:0031227	NA	1.00	**4.25**	**0.03**	intrinsic to endoplasmic reticulum membrane
GO:0003824	0.86	0.48	**0.82**	**0.05**	catalytic activity
GO:0016491	**2.69**	**0.02**	**1.94**	**0.03**	oxidoreductase activity
GO:0016712	**5.06**	**0.01**	3.37	0.28	oxidoreductase activity acting on paired donors with incorporation or reduction of molecular oxygen reduced flavin or flavoprotein as one donor and incorporation of one atom of oxygen
GO:0020037	**4.04**	**0.01**	1.93	0.72	heme binding
GO:0046906	**4.04**	**0.01**	1.93	0.72	tetrapyrrole binding
GO:0004497	**4.42**	**0.02**	2.31	0.59	monooxygenase activity

Gene Ontology (GO) analysis was used to determine if genes exhibiting Type-I (dioxin-responsive across both collectives) or Type-II (dioxin-responsive in only one collective) character were functionally coherent. Lists of Type-I and Type-II genes generated as described in the Results were tested for enrichment of each GO category represented on the RAE230A array. False-discovery rates (FDR) were calculated with 1000 permutations of the dataset using the High-Throughput GoMiner software, bolding highlights those terms with a FDR <5%. Enrichment values are expressed in log_2_-space (i.e. a value of 3.0 indicates an 8-fold enrichment). Note that GO:0016491 (oxidoreductase activity) is enriched in both the Type-I and Type-II gene lists.

## Discussion

We performed transcriptional profiling on livers of rats that are sensitive or resistant to major TCDD toxicities. Two key findings arise. First, we show significant inter-strain and inter-species diversity in responses to TCDD. Second, we identify Type-II genes that may be integral to the mechanism(s) of hepatotoxicity, wasting and lethality.

### Significant diversity of intra-species and inter-species responses to TCDD

One startling characteristic of the transcriptional response to TCDD across the four rat strains/lines are the dramatic inter-strain differences. Given their substantial genetic relatedness, including at the *AHR *locus, it might be hypothesized that LnA and H/W animals would have very similar responses, and that LnC would be very similar to L-E. To the contrary, 68.1% (308 of 452) of dioxin-responsive genes were altered in one of the four rat strains/lines. These results clearly demonstrate the importance of genomic context in regulating mRNA responses to dioxin-exposure and mirror an analysis of the basal mRNA levels in these and other rat strains (Boutros et al. submitted). Interestingly, in rats with the *AHR*^H/W ^genotype, the total number of genes that respond was reduced relative to rats expressing wildtype AHR. However, AHRs from rats with the *AHR*^H/W ^genotype have similar affinity for TCDD and ability to bind AH response elements as wildtype rats [36]. It is conceivable that *AHR*^H/W ^rats have a reduced ability to recruit coactivators and interact with transcriptional machinery. Chromatin immuno-precipitation experiments would be valuable in testing this hypothesis directly.

When we attempted to study AHR-dependency of the mRNA changes by comparison with *Ahr*^-/- ^mice we found that only 2 of 4 mRNA responses to TCDD, measured by RT-PCR, could be compared in both rat and mouse models. This result concords with recent reports of highly divergent transcriptomic responses to TCDD between rat and mouse [23], and suggests that combining our intra-species rat model with inter-species studies may be a fruitful approach for identifying genes that mediate TCDD-induced toxicities, especially those toxic responses that differ between animal species.

### Type-II responsive genes whose regulation by TCDD may be integral to the mechanism(s) of hepatotoxicity, wasting and lethality

In dioxin-sensitive rats, a wasting syndrome commences within the first few days following a single dose of TCDD and is characterized by progressive weight loss (eventually up to 50%) and hypophagia [37-39]. Wasting contributes to lethality starting 2-3 weeks after TCDD exposure. Although wasting accompanies death, wasting *per se *is not likely to be the sole reason for death since maintenance of body weight by parenteral nutrition does not prevent mortality [40]. Nevertheless, untreated control rats, pair-fed at the same caloric intake as rats treated with lethal TCDD doses, die at much the same time as their TCDD-exposed partners [41].

The cause of TCDD-induced wasting and ultimate death as well as the key target tissue(s) remain elusive. Since TCDD causes extensive hepatotoxicity in TCDD-sensitive rats (but not in the TCDD-resistant strains), it is reasonable that examination of mechanisms which produce hepatotoxicity may provide clues to mechanisms of wasting and lethality. To this end, we identified 46 Type-II hepatic genes whose TCDD-responsiveness differed between TCDD-resistant and TCDD-sensitive rats. Pathway analysis indicated that these genes are mainly involved in lipid-metabolism, cellular membrane function and energy metabolism (Table 4). These Type-II genes potentially explain why there are greater manifestations of hepatotoxicity in sensitive rats than in resistant rats; for example, a dramatic accumulation of fatty acids (steatosis) and initial liver hypertrophy which switches to atrophy ~1 week later (refer to [24] for exhaustive list of hepatotoxic responses). Pohjanvirta et al. [24], in biochemical studies, found that exposure of sensitive rats to TCDD led to steatosis, hypertrophy, liver failure, wasting and eventual death, possibly as the consequence of derailment of energy metabolism due to alterations of (i) lipid homeostasis, (ii) protein metabolism and/or (iii) ATP production/utilization.

#### (i) Alteration of lipid homeostasis

In sensitive rats only, we previously observed steatosis with the accumulated fatty acids probably originating from redistribution of peripheral fat deposits to liver rather than from increased lipid synthesis within the liver [42]. This is consistent with our current transcriptomic study in which transcripts related to lipid synthesis were not increased. TCDD-induced suppression of hepatic lipid lipogenesis previously has been reported [16,17,43].

Pathway analysis indicated that genes involved in the lipid metabolism process were enriched in the Type-II gene list. Of particular interest was the decreased expression of *Hsd11b1* and *Slc27a5* only in sensitive rats. *Hsd11b1* functions in steroid metabolism and colocalizes with the glucocorticoid receptor where it acts as a local amplifier of corticoid responses including the regulation of fuel metabolism during starvation and stress [44,45]. *Hsd11b1* deficiencies in rodents increase energy expenditure, decrease weight gain with chronic high fat feeding, increase weight loss, increase hepatic lipid oxidation while decreasing lipolysis in adipose tissue and display many metabolic deficiencies [45,46]. *Slc27a5* encodes a transporter of long-chain fatty acids into the liver where it is exclusively expressed. Its deletion results in increased de novo biosynthesis of long-chain fatty acids in liver due to inhibited uptake of them. Interestingly, in knockout mice, feed intake is depressed, energy expenditure increased and weight gain suppressed [47,48]. While further study of these genes is warranted, genes that alter lipid homeostasis may be important in hepatotoxicity and could be involved in pro-death pathways in sensitive rats exposed to TCDD.

#### (ii) Altered protein metabolism

In the short term, protein catabolism can be a beneficial response to provide amino acids for energy and maintenance of obligatory functions. However, sustained protein catabolism eventually leads to wasting and mortality. Our current study found that TCDD increased expression of genes that facilitate protein breakdown (*Derl1*, *Derl2 *and *Mug1*) but also decreased the expression of *Ass1*, the key enzyme in the urea cycle. Deficiencies in *Ass1* may disrupt the urea cycle resulting in increased accumulation of amino acids, highly toxic ammonia and other toxic byproducts. TCDD previously has been shown to decrease the expression of the *Ass1* gene after 24 hour exposure [17]. Increased protein breakdown and deficiencies in the urea cycle are consistent with previous reports of elevated plasma levels of most amino acids and decreased plasma urea in sensitive rats but not in resistant rats 6 days after TCDD exposure [49]. In addition, alteration of the balance between protein synthesis versus protein degradation is a key mechanism in switching hepatocytes from hypertrophy to atrophy, as observed in sensitive rats ~1 week after TCDD exposure [24] and reported for other wasting diseases (e.g. diabetes and cancer cachexia) [50]. Thus, specific genes involved in protein homeostasis are likely important in TCDD-mediated pro-survival pathways.

#### (iii) Impaired ATP production/utilization

Derailment of energy metabolism due to impaired ATP production or utilization potentially contributes to manifestations of major TCDD toxicities. After TCDD exposure in sensitive rats, compensatory mechanisms may attempt to increase energy for metabolism by increasing expression of the *Atp5c1 *gene that resides within the ATP synthesis pathway and is down-regulated in obese subjects [51]. However, TCDD decreased expression levels of *Adk* which catalyzes the inter-conversion of adenine nucleotides, and plays an important role in cellular energy homeostasis (2 ADP ↔ ATP + AMP); this downregulation may impair use of ATP as an energy source in sensitive rats. Specifically, *Adk* impairment leads to deficiencies in adenosine nucleotides, including ATP, likely leading to reduced mitochondrial metabolic capacity and impairment of lipid metabolism critical for energy production [52]. Moreover, *Adk*-deficient mice display hepatic steatosis within 4 days and die within 14 days with fatty liver [52]. Thus, deficiencies in adenosine metabolism are powerful contributors to development of hepatic steatosis and development of lethal fatty liver, processes that are also provoked by TCDD in sensitive rats.

In dioxin-resistant rats, where TCDD does not cause severe hepatotoxicity, wasting or death, there was no alteration of mRNA levels for genes which might derail energy metabolism due to alterations of in the homeostasis of lipids, protein metabolism or ATP production/utilization. Only three genes responded to TCDD in the resistant collective but not in the sensitive collective. Two of these three genes function in lipid metabolism: the expression of *Phyh* was increased while that of *Hacl1* was decreased. The third gene *Il1r1*, whose levels were increased by TCDD, is a receptor whose responsiveness regulates several biological functions, including adaptive and innate immunity, control of programmed cell death and stress response [53]. The consequences of altered regulation of these genes in mediating potential pro-survival pathways in response to TCDD warrants further investigation.

## Conclusion

The mechanisms of dioxin-induced toxicities remain elusive but our transcriptomic approach in an *in vivo *rat model where there are major phenotypic differences in the toxic response is providing clues to the early events that may trigger toxicity. Compelling evidence shows that the transcriptional activity of the AHR is essential for toxicity. Because altered transcription is central to TCDD toxicity, our group and others have profiled changes of mRNA abundance resulting from exposure to TCDD in several model systems. The results are remarkable: TCDD induces wide-spread alterations in mRNA abundance, but only a very small fraction of these changes are conserved between mouse or rat and, as demonstrated here, within different rat strains. Only 31.9% of dioxin-responsive genes are altered in more than one of the four rat strains/lines.

This diversity of transcriptional responses makes it challenging to identify specific genes responsible for lethality and other major forms of dioxin toxicity. Our results suggest that hepatic toxicity probably is not caused by dysregulation of a single critical gene. Rather, pathways such as lipid metabolism or energy metabolism may be derailed by altered transcription of multiple genes, possibly under coordinate control of the AHR with participation of other regulatory factors. Pathways and individual genes highlighted here are worthy candidates for further mechanistic studies to test their role in mediating or protecting from major dioxin toxicities.

## Methods

### Animals and Treatments for Rat Model

We studied two dioxin-sensitive rat strains/lines expressing wildtype AHR: Long-Evans (*Turku/AB*) (L-E) and Line-C (LnC). We also studied two dioxin-resistant rat strains/lines expressing the Han/Wistar variant AHR: Han/Wistar (*Kuopio*) (H/W) and Line-A (LnA) [25]. All animals were males 10-12 weeks of age from breeding colonies of the National Institute for Health and Welfare, Kuopio, Finland. They were housed in groups of 4 (an entire treatment group per cage) in suspended stainless-steel wire-mesh cages with pelleted R36 feed (Lactamin, Stockholm, Sweden) and tap water available *ad libitum*. The temperature in the animal room was 21 ± 1°C, relative humidity 50 ± 10%, and a 12 hour-light/12 hour-dark cycle. Study plans were approved by the Animal Experiment Committee of the University of Kuopio and the Provincial Government of Eastern Finland. There were four rats per treatment group. Liver was harvested between 8:30 and 11:00 from rats treated by gavage with a single 100 μg/kg dose of TCDD or corn oil vehicle 19 hours previously. The single dose of 100 μg/kg TCDD produces hepatotoxicity, wasting and death in sensitive rats but not in resistant rats.

### Animals and Treatment for Mouse Model

Liver tissues were from mice in which we previously mapped AHR-dependent and dioxin-dependent gene batteries by transcriptomic analysis [20]. Briefly, male *Ahr*-null (*Ahr^-/-^*) mice in a C57BL/6J background (10 weeks old) and C57BL/6 mice carrying wildtype (*Ahr^+/+^*) (15 weeks old) were given a single dose of 1000 μg/kg TCDD or corn oil vehicle by gavage. Liver was harvested 19 hours after treatment. The single dose of TCDD is lethal to wild-type but not *Ahr*-null mice and is equitoxic to that given to sensitive rats. We tested 3 TCDD-treated and 3 control mice in the *Ahr^-/- ^*groups and 4 TCDD-treated and 4 control mice in the *Ahr*^***+/+ ***^groups.

### RNA Extraction

Total RNA was extracted using Qiagen RNeasy kits according to the manufacturer's instructions (Qiagen, Mississauga, Canada). Total RNA yield was quantified by UV spectrophotometry and RNA integrity was verified using an Agilent 2100 BioAnalyzer (Agilent Technologies, Santa Clara, CA).

### Microarray analysis

Sample labeling and hybridization to Affymetrix RAE230A GeneChips^® ^were performed by The Centre for Applied Genomics (Toronto, Canada) according to the manufacturer's protocols. At each condition four separate animals were profiled, each on an individual RAE230A microarray. Raw array data were examined for spatial and distributional heterogeneity and differential RNA degradation; no arrays were excluded. Array data were loaded into the R statistical environment (v2.9.2) using the affy package (v1.22.1) of the BioConductor open-source library [54]. Array data were pre-processed with the RMA algorithm [55]. Raw and pre-processed array data are available in the Gene Expression Omnibus repository at NCBI (accession GSE10083). An alternative CDF package was used to ensure each ProbeSet was mapped to a single unique Entrez Gene ID (rae230arnentrezgcdf v12.0.0) [56].

### Statistical analysis of array data

The experimental design employed independent pair-wise analyses between treated and control animals for each strain/line (Figure 1). Following quality control and pre-processing of the microarray data, we performed a general linear modeling analysis. For each gene and each strain we determined the magnitude of differential signal intensity between TCDD-exposed and vehicle-treated animals. Gene-lists were derived separately for each strain using the limma package (v2.18.3) in the R statistical environment (v2.9.2) with a condition-specific design matrix and within-strain pair-wise contrasts. An empirical Bayes moderation of the standard error [57] and false-discovery rate control of multiple-testing were applied [58]. A significance threshold of p_adjusted _< 0.01 was applied to each contrast. We then scored each gene using a scheme described previously [30]. Briefly, each gene was classified as unaltered (Score: 0), statistically significantly repressed by TCDD (Score: -1), or statistically significantly induced by TCDD (Score: +1) in each strain, and these strain-wise scores were summed.

Unsupervised machine-learning was performed using divisive hierarchical clustering with complete linkage in the R statistical environment (v2.9.2) using the cluster package (v1.12.1). Pearson's correlation was used as a similarity metric and within-row scaling was performed. Venn diagrams were produced using custom R code. R visualizations employed the lattice (v0.17-26) and latticeExtra (v0.6-3) packages.

### Functional characterization of responsive genes

To determine if genes perturbed by TCDD are enriched for specific Gene Ontology (GO) terms, we first identified groups of genes dysregulated by TCDD within each strain/line (p_adjusted _< 0.01). Then GO terms enriched in these groups were identified using the GoMiner tool [59]. False-discovery rates (FDRs) were estimated using 1000 permutations. Rat-specific annotations were used. GO terms with false-discovery rates below 5% were included in subsequent analyses.

### mRNA quantitation by Real-Time RT-PCR

Total RNA (2 μg) was reverse-transcribed into cDNA using oligo-dT primer p(dT)15 (Roche Applied Science, Laval, QC, Canada) and Superscript II RNA polymerase according to the manufacturer's instructions (Invitrogen, Carlsbad, CA). Real-time PCR was performed using in-house designed primers (with 5' fluorogenic probes, as described previously [30]) or Applied Biosystems gene expression assays, as described by the manufacturer (Applied Biosystems, Forest City, CA). Primer/probe sequences are in Additional File 2.

Normalized expression was calculated as 2^−ΔΔ*C*t^, where *C*_t _is the threshold cycle for detecting fluorescence. PCR amplification efficiency was determined from a 10-fold serial dilution of a cDNA pool; efficiency ranged from 90-110% for all genes. Data were normalized to either Actb or Gapdh, genes we previously showed to be suitable as normalization standards for dioxin studies [60]. In the rat model, significant differences in mRNA levels were determined using t-tests (two-tailed, unequal variance). Significant differences in mRNA levels in the *Ahr*-null mouse model were identified using analysis of variance (ANOVA) with Bonferroni *post hoc *tests (GraphPad version 4.0).

## Authors' contributions

The project was conceived and designed by ABO and RP. Animal treatment was performed by RP. mRNA extraction and RT-PCR analyses were performed by IDM. Microarray data and functional analyses were performed by PCB. Microarray data visualization was performed by PCB and HC. IDM wrote the first draft of the manuscript, which PCB, ABO and RP edited and all authors approved.

## Supplementary Material

Additional file 1 **Complete Microarray Results**. This file gives a list of all genes interrogated on the microarray, their annotation, and their changes in signal-intensity in each rat strain. Click here for file

Additional file 2 **Primer and Probe Sequences**. This file lists all primer and probe sequences used for RT-PCR analyses. Click here for file
