# Supplementary material for: Aryl hydrocarbon receptor (AHR)-regulated transcriptomic changes in rats sensitive or resistant to major dioxin toxicities
Source: BMC Genomics. 2010 Apr 26;11:263. doi: 10.1186/1471-2164-11-263 (PMC2881023; doi:10.1186/1471-2164-11-263)
Supplement: Additional file 2 — Primer and Probe Sequences. This file lists all primer and probe sequences used for RT-PCR analyses. [file 1471-2164-11-263-S2.DOC]

| **Additional Table 2. Sequences of primers and probes used for real-time RT-PCR.** | | | |  | | |
| --- | --- | --- | --- | --- | --- | --- |
|  |  |  |  |  | | |
| **Gene Symbol** | **Accession Number** | **Forward Primer** | **Reverse Primer** | **Probe** | | **Size (bp)** |
| **5’fluorogenic probe** | |  |  |  | |  |
| *Actb* | NM_031144 | GACCCAGATCATGTTTGAGACCTTC | GGAGTCCATCACAATGCCAGTG | ACGACCAGAGGCATACAGGGACAACACAG | 110 | |
| *Chka* | NM_017127 | GTCATCAGGGGTGGTCTCAGTA | CCATACAGTCGCAAGAGCACTT | TGCTGTTCCAGTGTTCCTTGCCAGACT | 104 | |
| *Crip2* | NM_022501 | GCTGGTTCCTACATCTACGAGAAG | CACTAGAGGCTTTGCTGGGAC | CCGCTGGTCTTCCGCTCTTCAGTTCGTA | 133 | |
| *Cyp1a1* | NM_012540 | GAATGCCAATGTCCAGCTCTCA | TACCAGGTACATGAGGCTCCAA | AGCAGTTGTGATTGTGTCAAACCCAGCTCC | 115 | |
| *Cyp7a1* | NM_012942 | ATCTACCCAGACCCTTTGACTTTC | CACTTCAGCTTGTTTCCATTACTGTA | AGGTGGTCTTTGCTTTCCCGCTTTCATCA | 98 | |
| *Per2* | NM_031678 | GGCAGCCAAAGGCACCTC | CGCCCAGGAGGAGGTTGA | AACATGCAGTGAGCCCTCAGACACCCA | 99 | |
| *Selenbp1* | NW_047626 | AATGCCTGGTTTGATCACCGA | TAGAGATGTCATACTGCCGAATGT | TCTTGCTGTCCCTGGATGACCGCTT | 101 | |
| **ABI: gene expression assays Assay ID** | | | **Size (bp)** |  | | |
| *Elovl6* | NM_134383 | Rn00592815_m1 | 67 |  | | |
| *Gapdh* | NM_017008 | Rn99999916_s1 | 87 |  | | |
| *Klf10* | NM_031135 | Rn00579697_m1 | 78 |  | | |
| *Pik3r1* | NM_013005 | Rn00564547_m1 | 97 |  | | |
